# DIAGNOSIS AND TREATMENT OF SMALL INTESTINAL BACTERIAL OVERGROWTH: AN OFFICIAL POSITION PAPER FROM THE BRAZILIAN FEDERATION OF GASTROENTEROLOGY

**DOI:** 10.1590/S0004-2803.24612024-107

**Published:** 2025-02-17

**Authors:** Bruno César da SILVA, Gabriela Piovezani RAMOS, Luisa Leite BARROS, Ana Flávia Passos RAMOS, Gerson DOMINGUES, Décio CHINZON, Maria do Carmo Friche PASSOS

**Affiliations:** 1 Hospital da Bahia, Divisão de Gastroenterologia, Salvador, BA, Brasil.; 2 Faculdade Evangélica Mackenzie do Paraná, Divisão de Gastroenterologia, Curitiba, PR, Brasil.; 3 Faculdade de Medicina da Universidade de São Paulo, Departamento de Gastroenterologia, São Paulo, SP, Brasil.; 4 Santa Casa de Belo Horizonte, Divisão de Gastroenterologia, Belo Horizonte, MG, Brasil.; 5 Universidade do Estado do Rio de Janeiro, Rio de Janeiro, RJ, Brasil.

**Keywords:** Small intestinal bacterial overgrowth, SIBO, breath test, Supercrescimento bacteriano no intestino delgado, SIBO, teste respiratório

## Abstract

**Background:**

Small intestinal bacterial overgrowth (SIBO) is a condition characterized by an abnormal increase in bacterial population in the small intestine, leading to symptoms such as bloating, abdominal pain, distension, diarrhea, and eventually malabsorption. The diagnosis and management of SIBO remain challenging due to overlapping symptoms with other gastrointestinal disorders such as inflammatory bowel disease (IBD), irritable bowel syndrome (IBS), and coeliac disease.

**Objective:**

This article aims to review current evidence on the diagnosis and treatment of SIBO, with a focus on strategies suitable for the Brazilian healthcare system.

**Methods:**

A comprehensive literature review was performed, focusing on clinical guidelines, randomized controlled trials, and cohort studies concerning SIBO. Diagnostic methods, including breath tests and direct aspiration techniques, were critically analyzed. Treatment approaches, including antibiotics, dietary modifications, and probiotics, were reviewed. The recommendations were formulated based on a panel of gastroenterologists, members of the Brazilian Federation of Gastroenterology (FBG), with approval from the majority of the members.

**Results:**

Breath tests using glucose and lactulose remain the most commonly used non-invasive diagnostic tools, though they are subject to limitations such as false positives and false negatives. Treatment with rifaximin is effective in most cases of SIBO, while systemic antibiotics like metronidazole and ciprofloxacin are alternatives. Probiotics and dietary interventions, particularly low FODMAP diets, can complement antibiotic therapy. Long-term follow-up is essential due to the recurrence rate, which is common in SIBO patients.

**Conclusion:**

Standardizing SIBO diagnosis and treatment in Brazil is essential to reduce diagnostic delays and optimize care, especially given the disparities and heterogeneity in clinical practice across the country. This article provides evidence-based recommendations to guide clinical practice. Further research is needed to refine diagnostic methods, explore novel treatment strategies, and better understand the specific characteristics of the Brazilian population.

## HIGHLIGHTS

• A comprehensive review of clinical guidelines, randomized trials, and cohort studies on small intestinal bacterial overgrowth (SIBO) was built.

• Diagnostic tools analyzed include breath tests (glucose and lactulose) and direct aspiration techniques, both of which have the potential for false positives/negatives.

• The preferable treatment is Rifaximin, the most effective antibiotic for SIBO. Alternatives include metronidazole and ciprofloxacin.

• Standardization of SIBO diagnosis and treatment in Brazil is critical to reducing delays and improving care despite regional disparities. Further research is needed to refine diagnostic methods and explore treatment options specific to the Brazilian population.

## RESUMO

**Contexto:**

O supercrescimento bacteriano no intestino delgado (SIBO) é uma condição caracterizada por um aumento anormal da população bacteriana no intestino delgado, levando a sintomas como dor e inchaço abdominal, diarreia e, eventualmente, má absorção. O diagnóstico e o manejo do SIBO permanecem desafiadores devido à sobreposição de sintomas com outros distúrbios gastrointestinais, como doença inflamatória intestinal (DII), síndrome do intestino irritável (SII) e doença celíaca.

**Objetivo:**

Este artigo tem como objetivo revisar as evidências atuais sobre o diagnóstico e o tratamento do SIBO, com foco em estratégias adequadas ao sistema de saúde brasileiro.

**Métodos:**

Foi realizada uma revisão abrangente da literatura, com ênfase em diretrizes clínicas, ensaios clínicos randomizados e estudos de coorte relacionados ao SIBO. Métodos diagnósticos, incluindo testes respiratórios e técnicas de aspiração direta, foram analisados criticamente. Abordagens terapêuticas, como antibióticos, modificações dietéticas e probióticos, foram revisadas. As recomendações foram formuladas com base em um painel de gastroenterologistas, membros da Federação Brasileira de Gastroenterologia (FBG), com aprovação da maioria dos membros.

**Resultados:**

Os testes respiratórios com glicose e lactulose continuam sendo as ferramentas diagnósticas não invasivas mais utilizadas, embora apresentem limitações como falsos positivos e falsos negativos. O tratamento com rifaximina é eficaz na maioria dos casos de SIBO, enquanto antibióticos sistêmicos como metronidazol e ciprofloxacino são alternativas. Probióticos e intervenções dietéticas, especialmente dietas com baixo teor de FODMAP, podem complementar a terapia antibiótica. O acompanhamento de longo prazo é essencial devido à alta taxa de recorrência, que é comum em pacientes com SIBO.

**Conclusão:**

Padronizar o diagnóstico e o tratamento do SIBO no Brasil é essencial para reduzir atrasos diagnósticos e otimizar os cuidados, especialmente considerando as disparidades e a heterogeneidade na prática clínica no país. Este artigo apresenta recomendações baseadas em evidências para orientar a prática clínica. Mais pesquisas são necessárias para aprimorar os métodos diagnósticos, explorar novas estratégias de tratamento e compreender melhor as características específicas da população brasileira.

## INTRODUCTION

Small intestinal bacterial overgrowth (SIBO) is defined as an excessive bacterial population in the small intestine, representing a specific form of small intestinal dysbiosis. This condition is characterized not only by an abnormally high microbial density but also by the presence of microbe types typically found in the large intestine. The resultant dysbiosis leads to a range of symptoms, including diarrhea, bloating, and abdominal pain, which significantly impair the quality of life of those affected^1^. The impact of SIBO on patient health is profound. Affected individuals may suffer from malnutrition, weight loss, and various nutrient deficiencies, complicating the clinical picture and often resulting in symptoms that can be mistaken for other gastrointestinal conditions such as irritable bowel syndrome (IBS)^2^ ^,^ ^3^.

Diagnostically, the traditional threshold for defining SIBO in the jejunal aspirate has been set at ≥10^5^ CFU/mL^4^. However, a more recent threshold of ≥10³ CFU/mL has been adopted for duodenal aspirate^5^. These criteria are important for accurately identifying and managing the condition based on the specific site and bacterial population within the small intestine. Despite jejunal aspirate being the gold standard for diagnosis, breath testing has been widely adopted due to its less invasive nature and greater availability in healthcare settings.

In Brazil, the relevance of SIBO in gastroenterology is increasingly recognized due to its potential widespread impact, particularly among vulnerable populations such as the elderly and those with predisposing gastrointestinal conditions. Despite this growing awareness, there is a significant gap in both clinical practice and research within the country. Notably, there is a marked scarcity of local epidemiological data, with few studies specifically investigating SIBO in the Brazilian population. The absence of standardized guidelines can result in heterogeneity in treatment protocols and disparities in care across different institutions and clinicians. The absence of uniform guidelines and empirical data may result in varied treatment outcomes and care disparities across healthcare settings.

The objective of this position paper is to shed light on the definition and diagnostic criteria for SIBO and propose standardized a Brazilian guidelines for its management. Enhanced national coordination and adherence to these recommendations are intended to promote better clinical outcomes, ensure a uniform standard of care, and fulfill an urgent need in Brazilian gastroenterology, thereby improving the quality of life for patients.

## PATHOPHYSIOLOGY AND RISK FACTORS

### Pathophysiological mechanisms of SIBO

The pathophysiology of SIBO involves the interplay between bacterial overgrowth and the host’s immune and digestive systems. Overgrowth leads to fermentation of undigested carbohydrates, resulting in the production of gases like hydrogen and methane, which contribute to symptoms such as bloating and abdominal discomfort. Additionally, bacterial metabolites can cause direct mucosal injury, leading to malabsorption and nutrient deficiencies^1^.

### Risk factors for SIBO

The small intestine typically harbors relatively few bacteria due to the hostile environment created by various physiological processes. Factors that maintain low bacterial levels include gastric acid, pancreaticobiliary secretions, local immunity, and the normal structure and function of the small bowel, particularly the peristaltic activity which sweeps bacteria toward the colon. Disruption of these mechanisms can predispose individuals to SIBO through several pathways:

**Gastrointestinal motility disorder**: under normal conditions, the gastrointestinal system coordinates complex smooth muscle contractions known as the migrating motor complex (MMC), which propels food and debris through the GI tract during fasting and prevents bacterial stagnation. Decreased MMC activity, influenced by factors such as medication use (e.g., opioids) or intrinsic damage to enteric nerves and muscles (common in conditions like diabetes or connective tissue disease), can significantly increase the risk of SIBO^6^.

**Abnormal gastrointestinal secretory function**: the acidity of the stomach, which ranges from pH 1 to 3, forms a critical barrier against bacterial colonization. Reduced acid production, whether due to the use of proton pump inhibitors (PPIs) or conditions such as hypochlorhydria, can facilitate bacterial overgrowth. Similarly, impaired pancreatic function reduces the secretion of key digestive enzymes that also possess antibacterial properties, further predisposing individuals to SIBO^7^ ^,^ ^8^.

**Structural alterations:** congenital or acquired abnormalities in the structure of the small intestine can lead to SIBO. Examples include strictures, diverticula, and surgical alterations like those seen in Roux-en-Y gastric bypass, which create environments conducive to bacterial stasis and overgrowth^9^.

**Dysfunctional gut immunity:** the gut immune system, comprising immune cells and barriers like secretory IgA and antimicrobial peptides, plays a crucial role in maintaining microbial balance. Dysfunction in these immune components can lead to an increased risk of SIBO^10^.

The diseases and disorders that has an association with SIBO are presented on Table 1.

**TABLE 1:** SIBO risk factors.

Pathophysiology	Condition
Small intestine dysmotility	Diabetic neuropathy
	Systemic sclerosis
	Amyloidosis
	Hypothyroidism
	Idiophatic intestinal pseudo-obstruction
	Gastroparesis
	Chronic opioid use
	Long-standing use of motility suppressing agents
Anatomic abnormalities	Small intestines diverticulosis
	Surgically induced alterations in anatomy (eg. Bypass)
	Strictures (eg: Crohn’s disease)
	Blind loops
	Fistulas
	Ileocecal valve ressection
Immune deficiency	Inherited immune deficiencies
	Aquired immune deficiencies (AIDS, severe desnutrition)
Multifactorial	Diabetes Mellitus
	Chronic pancreatitis
	Cystic fibrosis
	Celiac disease
	Crohn’s disease
	Radiation enterophaty
	Liver diseade
	End-stage renal disease
	Senility
Unclear relationship	Rosacea
	Intestitial cystitis
	Restless legs syndrome
	Parkinson’s disease
	Severe obesity
	Irritable bowel syndrome

**Statement 1**: several factors increase the risk of SIBO, including gastrointestinal motility disorders, reduced gastric acid secretion, structural abnormalities in the small intestine, and immune deficiencies. Conditions such as diabetes, systemic sclerosis, Crohn’s disease, and chronic opioid use, among others, are commonly associated with these risk factors, predisposing individuals to bacterial overgrowth.

### Interaction between SIBO and other gastrointestinal disorders

SIBO frequently coexists with other gastrointestinal disorders such as irritable bowel syndrome (IBS), inflammatory bowel disease (IBD), and celiac disease^3^ ^,^ ^11^ ^,^ ^12^. This coexistence can complicate the diagnosis and management of these conditions, as SIBO can mimic or exacerbate their symptoms. For instance, the inflammatory changes in Crohn’s disease can disrupt normal gut motility and anatomy, thus facilitating bacterial overgrowth. Moreover, the altered motility and immune function seen in systemic diseases like diabetes and scleroderma also increase the risk of developing SIBO, thereby creating a complex interplay of factors that need careful consideration during diagnosis and treatment^10^.

## EPIDEMIOLOGY

### Prevalence of SIBO globally and in Brazil

The global prevalence of SIBO varies widely, reported between 2.5% and 22%, with higher rates observed in older adults and individuals with comorbid conditions^13^. In Brazil, data are sparse, yet some studies have begun to shed light on SIBO’s local impact. For instance, a study by Bertges et al. reported a 30% prevalence in Brazilian Crohn’s disease patients^14^, while Martins et al. found significant prevalence rates of 56% and 64% in patients with gastrointestinal symptoms using hydrogen and methane breath tests, respectively^15^. These findings highlight the variability and the influence of local demographic and disease-related factors compared to data from other regions.

### Challenges in epidemiological studies of SIBO

Epidemiological research on SIBO faces challenges due to the variability in diagnostic criteria and testing methods. The use of different breath tests, such as glucose and lactulose, can result in varied prevalence rates^16^. Furthermore, symptom overlap with other gastrointestinal disorders complicates the diagnosis^17^. Additionally, the necessity for dual gas detection in breath tests (for hydrogen and methane) is crucial for accuracy, as methanogenic bacteria do not produce hydrogen^5^. These methodological issues underscore the need for standardized diagnostic criteria and improved testing techniques to better understand and manage SIBO, especially in regions like Brazil where specific research is still developing.

### Clinical presentation

The clinical manifestations of SIBO are broad and nonspecific. They range from symptoms such as bloating, distension, flatulence, abdominal pain, diarrhea, constipation to more severe manifestations, including weight loss or inability to gain weight, nutritional deficiencies, steatorrhea, anemia, and neuropathy^18^. For example, bacteria involved in bile acids metabolism could lead to malabsorption of fat and fat-soluble vitamins. On the other hand, microorganisms that produce folic acid and vitamin K may result in increased blood levels of these nutrients. Among the symptoms, diarrhea, rather than bloating, has the strongest association with SIBO^4^. Some studies have described a syndrome of brain fog and symptoms of anxiety and depression linked to SIBO, although it is unclear whether this is directly related to the condition^19^ ^,^ ^20^.

As the frequency of each nonspecific symptom varies among patients, diagnosing SIBO based on symptoms alone is very difficult. The diagnosis should be suspected, specially in at-risk population: those with motility disorders, medications affecting gut motility, surgically altered GI anatomy, immunodeficiencies, or altered GI mucosal secretion or gut barrier function^4^ ^,^ ^21^.

**Statement 2:** SIBO presents with nonspecific symptoms, including bloating, abdominal pain, diarrhea, and weight loss, with diarrhea having the strongest association. It should be suspected, especially in patients with motility disorders, altered GI anatomy, immunodeficiencies, or related conditions.

### Diagnosis

Due to the scarce specificity of symptoms for SIBO, the diagnosis requires objective tests for confirmation. Invasive and non-invasive methods are available, depending on accessibility, accuracy, and cost. Despite the gold standard diagnostic test being based on the culture of small bowel aspiration, due to its limitations, breath tests with lactulose and glucose are increasingly used worldwide. However, the lack of standardization in the protocol for performing these tests affects the interpretation of the results and, consequently, the correct diagnosis, epidemiology, and indication for treatment. Since there is no strong consensus on the appropriate diagnostic test for SIBO, efforts are ongoing to establish a standard protocol to be followed by all centers performing these tests. 

### Small bowel aspirate

Some recent evidence supports that small bowel aspiration remains the best technique for diagnosing SIBO, despite the lack of standardization of aspiration and microbiological techniques. Variability in the technique is based on the availability of required materials in different locations globally. In culture-based diagnostic testing, aseptic technique is critical to minimize the potential limitations of the test^18^.

Recently, a group of endoscopist from Georgia published the Rao technique for small bowel aspiration, attempting to standardize the methodology (Table 2)^22^. The current best method for SIBO investigation is theoretically the culture of a small bowel aspirate (jejunum) obtained during enteroscopy^23^. A bacterial concentration of ≥10^5^ colony-forming units per mL (CFUs/mL) in a jejunal aspirate culture is diagnostic of SIBO^10^. For duodenal aspirate culture, the threshold of ≥10^3^ CFUs/mL is used. The North American consensus recommended a minimum cutoff of 10^3^ CFUs/mL^17^. The most commonly identified species are Bacteroides, Enterococcus, and Lactobacillus^1^ *.*

**TABLE 2:** Rao technique description to perform a small bowel culture aspiration.

Steps	Description
Materials needed	Upper endoscopy 6F Liguory catheter *(COOK Medical, Bloomington, Ind, USA)*
	Sterile gloves / Sterile cap / 5-mL sterile syringe.
Initial test preparation	Prepare the catheter assembly and aspiration kit using sterile gloves.
Introduction of endoscope	A sterilized upper endoscope, flushed with sterile water before intubation, is passed into the small bowel with minimal air insufflation.
Catheter insertion	Endoscopist staff changes to sterile gloves during specimen collection. Then, he passes the Liguory catheter through the biopsy channel of the scope, using a short overtube to prevent valve contamination.
Aspiration	1. The assistant managing the syringe typically sits for gravity-assisted suction, gently aspirating fluid by repeated suctions with a 5 mL syringe connected to a 3-way stopcock.
	2. Between 3 to 5 minutes, 2-5 mL of bile-stained small bowel juice is successfully aspirated. (If aspirate collection is delayed, massaging the liver area can facilitate bile flow into the intestinal lumen).
	3. The syringe is capped with a sterile cap, immediately placed in a biohazard bag, and sent to the microbiology laboratory for aerobic and anaerobic cultures.

Although it is considered the gold standard diagnostic approach, it has several drawbacks: it is invasive, expensive, time consuming, and not available worldwide^1^ ^,^ ^23^ ^,^ ^24^. In addition, the technique predisposes to both false positives (due to contamination from oropharyngeal and gastric bacteria) and false negatives (due to the irregular distribution of bacteria in the bowel, leading to a non-representative samples). A recent study showed that using a single-lumen catheter to aspirate small bowel fluid resulted in a 19.6% contamination rate^25^. Furthermore, only 40% of the total gut flora can be identified using conventional culture methods^26^. Not all hydrogen-producing bacteria are culturable, and some microbiology laboratories are not able to culture methanogenic archaea^27^. A meta-analysis of culture methods showed that other gastrointestinal disorders can also increase small bowel bacteria counts besides SIBO^28^.

Statement 3: small intestinal aspirate remains the gold standard for SIBO diagnosis. While jejunal aspirate with ≥10^5^ CFU/mL has been the traditional threshold, duodenal aspirate with the updated threshold of ≥10³ CFU/mL is now recognized as the new reference standard for diagnosis.

### Breath tests

### Overview

Healthy individuals typically have approximately 100 milliliters of intestinal gas, primarily hydrogen (H_2_), carbon dioxide (CO_2_), and methane (CH_4_). There are four main sources of intestinal gas production: swallowed air; chemical reactions in the gut; diffusion of gases from the bloodstream; and microbial metabolism^29^. It has recently been demonstrated that the gut bacteria can be grouped into hydrogen producers and hydrogen consumers, which include methanogens (archaea) and sulfate-reducing bacteria that produce hydrogen sulfide gas (H2S)^30^ ^,^ ^31^. The gases resulting from microbial fermentation of carbohydrates in the gut include H_2_, CH_4_ and H_2_S. These gases diffuse into the abdominal venous circulation and are transported to the lungs, where they can be detected in exhaled breath^29^ ^,^ ^32^. 

Approximately 30% of the adult gut microbiome consists of methane producers, which can lead to false-negative results in hydrogen production and breath excretion^33^. Adding methane measurements to the hydrogen breath test has been proposed to improve SIBO diagnosis by approximately 20-30% in affected patients^34^. The additional measurement of H_2_S is still under development and is not yet widely commercially available, although it has the potential to further enhance the test’s reliability in diagnosing SIBO^31^.

To conduct a breath test (BT), the fasted subject ingests a carbohydrate substrate, and breath samples are obtained to measure the production of intestinal gases. There are two types of equipment available worldwide: one that measures only H_2_, and the other that measures both H_2_ and CH_4_ ^5^. Unfortunately, until the moment this manuscript was written, experience with equipment that measures CH_4_ in Brazil is limited, and it is not yet commercially available.

Although it is safe, non-invasive, and cheaper than culture of small bowel aspirate, there is no standardized methodology for breath testing. In special because there is more than one protocol developed by different consensus groups around the world. There is limited agreement regarding the published BT guidelines. The consensus from the German Society and Italian Society, published in 2005 and 2009 respectively, did not include methane measurement or consider the advancements in microbiome research from recent years^35^ ^,^ ^36^.

Based on this, the North American consensus attempted to incorporate as many key points and pieces of evidence as possible to create an easy-to-follow guideline for performing a BT^5^. In the past few years, an American College of Gastroenterology Clinical Guideline and an European Guideline from the European Association for Gastroenterology, Endoscopy and Nutrition, the European Society of Neurogastroenterology and Motility, and the European Society for Paediatric Gastroenterology Hepatology and Nutrition have been released^1^ ^,^ ^37^.

While there is no universal protocol for BT, the North American Consensus is the most widely followed worldwide and seeks to establish a standard for physicians to both perform and interpret these tests. 

**Statement 4:** the breath test is the initial method of choice for SIBO diagnosis due to its non-invasive nature, practicality, and accessibility in routine clinical practice.

### Preparation for the BT

The BT relies on gas production resulting from bacterial fermentation of the substrate in the small intestine. To reduce the presence of gases related to the metabolism of substrates from other dietary sources and to minimize the effects of medications and lifestyle factors that may alter the results, proper preparation before the test is essential. 

### A) Diet and lifestyle factors

On the day before the test, it is necessary to avoid foods containing poorly absorbed, fermentable carbohydrates and dietary fibers. Most authors recommend performing the test in the morning after a minimum of an 8-hour fast. Smoking, chewing gum, and consuming candies should be avoided on the day of the test, as well as physical activity that may lead to hyperventilation.

### B) Medication

Multiple medications can influence intestinal transit time and affect test interpretation. If possible, and at the clinician’s discretion, they should be discontinued prior to the test. 

Prokinetics and laxatives should be stopped at least 24h before the test, according to the European Guidelines, and at least one week before, according to the North American Consensus^5^ ^,^ ^37^. Opioids, which slow down transit time, should be avoided the day before the test. If discontinuation is not feasible, the results should be interpreted with caution^5^.

Antibiotics can significantly alter the microbiota and hydrogen production, and they should be discontinued 4 weeks before the test. Although there is limited evidence regarding the potential interference of prebiotics and probiotics, it is recommended to avoid their use 24 hours prior to the test^1^ ^,^ ^5^ ^,^ ^37^.

Bowel preparations and colonoscopy should be avoided for at least 2 weeks before the test, and it is not necessary to discontinue the use of proton pump inhibitors^1^ ^,^ ^5^ ^,^ ^37^. 

The recommendations for BT preparation are summarized on Table 3. 

**TABLE 3:** Instructions that must be followed by patients before performing a breath test for SIBO.

Preparation	Explanation
Antibiotics should be avoided 4 weeks before BT	Alter H_2_ and CH_4_ composition on exhaled breath^38^.
Prokinetics drugs and laxatives should be avoided at least 1 week before BT.	In addition to modify microbiome production of gas, it can directly affect intestinal transit time and the substrate would get faster to the colon, increasing false positive results.
	If patient could not stand without these medications for a month before BT, it is strongly recommending the discontinuation at least for one week^39^.
Diet 24 hours before	Avoid fermentable complex carbohydrates and dairy products, including lactose and fructose diet^5^ ^,^ ^40^ ^,^ ^41^.
Fasting for 8 to 12 hours before	
Avoid smoking the day before	Increase H_2_ levels on exhaled breath and gastric emptying time^42^ ^,^ ^43^.
Do not perform intensive physical activities the day before and during the test	Hyperventilation could inversely affect H_2_ levels^44^.
On the day of the test, it is recommended mouth washing with antiseptics	Avoid oropharyngeal flora contamination^35^.

SIBO: small intestinal bacteria overgrowth; BT: breath test; H2: hydrogen; CH4: methane.

### The choice of the substrate

Two substrates can be used for the breath test: glucose and lactulose. Both are available worldwide and has its pros and cons that will be discussed in this section.

Glucose is rapidly absorbed in the proximal small bowel, potentially preventing the diagnosis of SIBO in the distal intestine when compared to lactulose^45^. Lactulose, a non-absorbable sugar, passes through the entire small bowel, identifying SIBO along the entire length of the intestine. However, lactulose results can often be affected by gut motility, especially in patients with rapid intestinal transit, with or without diarrhea, which may impact the BT results by mimicking colonic fermentation^46^. A recent meta-analysis of 14 studies comparing BT with lactulose and glucose to intestinal aspirate showed that glucose BT tends to perform better than lactulose BT, with sensitivities of 54.5% and 42%, and specificities of 83.2% and 70.6%, respectively. A limitation of this meta-analysis is the absence of a direct head-to-head comparison of glucose and lactulose BT, which may hinder the conclusion of whether glucose BT is truly superior^47^. Nevertheless, lactulose BT still has some advantages. For example, it is a useful alternative in diabetic patients, as glucose can result in hyperglycemia, which may secondarily impact test results. It could also be preferred in patients with slower GI transit, although this point remains unproven^1^ ^,^ ^48^.

The North American Consensus for BT recommends administering 75 g of glucose, taken with or followed by 250 mL of water, and 10 g of lactulose diluted in 250 mL of water. The European Guidelines suggest administering 50 g of glucose and 10 to 20 g of lactulose^5^ ^,^ ^37^. A recent UK study compared different substrate doses for SIBO testing (16 g vs 10 g of lactulose and 50 g vs 75 g of glucose). The results showed that 10 g of lactulose significantly reduced positive SIBO results compared to 16 g and induced more symptoms. A possible explanation is that a higher dose of lactulose accelerates intestinal transit, leading to more false-positive results. Regarding the comparison of glucose doses, 75 g of glucose significantly increased positive results compared to 50 g without provoking additional symptoms. Since glucose is absorbed in the proximal bowel and is not fermented by colonic bacteria, the positive results are likely to be true positives. Therefore, the 75 g dose is recommended in alignment with the North American Consensus^49^. 

### How to perform

The BT is usually performed in the morning after an 8 to 12-hour fasting period. First, a baseline breath sample is collected, followed by the administration of the substrate in a single bolus over a brief period. Breath samples are then collected every 15 to 20 minutes for 90 to 120 minutes, measuring both hydrogen and methane levels^1^ ^,^ ^5^ ^,^ ^35^ ^,^ ^37^. Since methane measurement is not yet available in Brazil, we strongly recommend evaluating symptoms during and after the BT to account for potential methanogenic flora.

Elevated levels of hydrogen (>20 ppm) and methane (>10 ppm) at baseline are typically due to poor compliance with the recommended diet on the day before the test, suggesting ongoing fermentation. However, these elevated levels may also reflect other factors such as the presence of foregut dysmotility or oral bacteria. The European Guidelines recommend using an oral antiseptic (chlorhexidine 10%) before testing, whereas the North American Consensus and ACG guidelines do not specify its necessity^1^ ^,^ ^5^ ^,^ ^37^. 

Nocturnal hypoventilation can cause slightly elevated fasting levels of breath H_2_, produced by persistent fermentable substrates in the colon. A recent study from Spain found that light walking for an hour before the test may help reduce high baseline hydrogen and methane levels^50^. A reasonable recommendation would be to consider the use of antiseptic and suggest light walking, not for all patients, but for those with elevated baseline hydrogen levels (>20 ppm), as this may allow the BT to be performed without rescheduling, thus reducing diagnostic delays.

The accuracy of the hydrogen BT is limited by several factors, including those mentioned above, but particularly by the orocecal transit time. The addition of scintigraphy, an independent measurement of transit time, could enhance the sensitivity of the test. However, this technology, along with CO_2_ monitoring (proposed to ensure adequate breath sample collection), is not available in Brazil^37^ ^,^ ^51^.

At the end of the BT, the patient is required to complete a symptom questionnaire, detailing the intensity of symptoms, which is essential for interpreting the results in conjunction with the hydrogen and methane breath curves.

### Breath test diagnostic criteria for SIBO

The diagnosis of SIBO requires a rise in hydrogen of at least 20 ppm from baseline, or a rise of ≥10 ppm in methane from baseline, or a combined rise in hydrogen and methane of ≥15 ppm from baseline within 90 minutes, according to the North American Consensus and Guidelines^1^ ^,^ ^5^.

The European Guidelines, however, suggest that a rise in hydrogen of 10-12 ppm is the most commonly used cutoff value for test positivity^37^. A recent meta-analysis comparing the performance of glucose and lactulose BT with jejunal aspirate culture demonstrated that the glucose BT, when a lower cutoff (less than >20 ppm) was used, showed slightly better performance, with a sensitivity of 61.7%, specificity of 81.6%, and an AUC of 0.79, compared to a sensitivity of 47.3%, specificity of 80.9%, and an AUC of 0.7 when the >20 ppm cutoff was applied^47^.

The interpretation of a breath test with baseline H_2_ >20 ppm, despite fasting, adherence to a pre-test diet, and mouth washing with an oral antiseptic, remains unclear. Further studies are needed to clarify whether this represents a variant of SIBO. Additionally, more research is necessary to determine the role of the breath test in assessing SIBO after therapy. 

Breath testing is a useful, safe, simple, and noninvasive tool for diagnosing SIBO and is widely used in clinical practice. The most important factor guiding its optimal performance is the pretest probability, which helps identify patients most likely to benefit from it. It is also crucial to use the best available protocol. Table 4 provides suggestions on how the breath test should be conducted in our clinical practice.

**TABLE 4:** Step-by-step guide for performing BT.

Step	Description and comments
Preparation	1.Day before test: Follow all the instructions (Table 3) before BT.
Performing BT	
Technique for H2 acquisition	Inhale deeply and hold it for 20 seconds. Then, instruct patient to place mouth on the mouthpiece of the equipment and exhaled air for at least 10 to 15 seconds.
Steps of the test	1.Baseline measurement
	2.Drink substrate chosen by physician
	3.Perform gas acquisition every 15 minutes after finished substrate ingestion until 90 minutes.
	4.Symptoms should be evaluated during the test.
Key recommendations	Baseline levels should not be higher than 10 ppm. Check if pre-test diet and instructions were followed by patient. Consider walking and if the patient performed mouth washing. High baseline value can represent GI dysmotility.

BT: breath test; H2: hydrogen; ppm: parts per million.

**Statement 5**: both glucose and lactulose are viable first-line substrates for the breath test. Glucose is preferred for its greater specificity in diagnosing proximal SIBO, while lactulose, despite a higher likelihood of false positives, is useful when distal SIBO is suspected. The choice of substrate should be tailored at the physician’s discretion for optimal accuracy.

**Statement 6:** the breath test has to be performed over 90 minutes, with gas measurements taken every 15 minutes. A rise of 20 ppm or greater in hydrogen (H_2_) or 10 ppm or greater in methane (CH_4_) from baseline is considered indicative of a positive result for SIBO. Key clinical features and diagnostics of SIBO are summarized in TABLE 5.

**TABLE 5:** Key points of SIBO clinical features and diagnosis.

1. The clinical spectrum of SIBO range from mild to moderate nonspecific gastrointestinal symptoms to more severe clinical consequences, such as nutritional deficiencies and weight loss.
2. SIBO should be considered in the presence of non-specific gastrointestinal symptoms and/or signs of malabsorption, in the absence of another diagnosis on image and endoscopy, especially in those with risk factors.
3. Although less specific and sensitive breath tests are the first line investigation due to it noninvasive technique, low price and accessibility worldwide.
4. Culture of small bowel aspirate is the gold standard diagnostic method. However, it is invasive and operator dependent (perfect technique).

### Treatment of SIBO

The initial management of SIBO should include the identification and correction of the potential cause. Additionally, supplementation of vitamin deficits, such as fat-soluble vitamins, vitamin B12 and assessing malnutrition is recommended. 

Antibiotics are currently the cornerstone in SIBO treatment, and they act by modulating small intestine microbiota reducing gastrointestinal symptoms. A recent meta-analysis evaluated symptomatic responses to antibiotics compared to no antibiotics in patients with SIBO. Six studies and 196 patients were included in the analysis. Patients were treated with rifaximin, norfloxacin and neomycin, or placebo for 7-14 days. Overall, the RR (95%CI) of improvement was 2.46 (1.33-4.55), with antibiotics, and the pooled response rate was 49.5% vs 13.7% for those treated with antibiotics compared with no antibiotics. The number needed to treat (NNT) for antibiotics to relieve symptoms in SIBO was 2.8, favoring the use of antibiotics. There was also no difference in RR when stratified the duration of treatment for 7 or 10 days^52^. However, there are limited data comparing the efficacy of different antibiotics, and most of the evidence derives from IBS studies. 

The available guideline of SIBO by the American College of Gastroenterology, published in 2020, recommends the objective diagnosis of SIBO prior to empiric treatment. The widespread use of antibiotics has raised concerns regarding the emerging of multidrug-resistant bacteria, intrinsic adverse events, and an increased risk of *Clostridioides difficile* infection in this scenario^1^. TABLE 6 outlines the key antibiotics and their respective doses recommended for treating SIBO.

**TABLE 6:** Antibiotics for the treatment of Small Intestine Bacterial Overgrowth (10 to 14 days).

Antibiotics	Dose
Amoxicillin-clavulanic acid	875/125 mg 3 times/day
Ciprofloxacin	250-500 mg 2 times/day
Doxycyclin	100mg 2 times/day
Metronidazole	250 mg 3 times/day
Neomycin	500 mg 2 times/day
Norfloxacin	400 mg daily
Rifaximin	550 mg 3 times/day
Tetracycline	250 mg 4 times/day
Trimethoprim-sulfamethoxazole	800/160 mg 2 times/day

### Systemic antibiotics

In the literature, the most common systemic antibiotics evaluated were amoxicillin-clavulanic acid, ciprofloxacin, doxycycline, metronidazole, neomycin, norfloxacin, tetracycline and trimethoprim-sulfamethoxazole. Numerous small trials have tested antibiotic regimens and they have shown effectiveness in reducing IBS and SIBO symptoms^1^. Among these, ciprofloxacin was one of the most studied medications. For instance, a study conducted in 2005 assessed the effects of ciprofloxacin on 12 patients with non-alcoholic steatohepatitis. After 5 days of treatment with ciprofloxacin 500 mg twice daily, small intestinal bacterial overgrowth (SIBO) was suppressed in 83% of the patients, as confirmed by the glucose hydrogen breath test (*P*=0.025)^53^.

Another study investigated the use of ciprofloxacin in 29 patients with Crohn’s disease and bacterial overgrowth. Treatment with ciprofloxacin 500 mg twice daily for 10 days led to breath test normalization in 100% of the patients. Symptom improvement was reported in 83% of cases for bloating, 50% for stool softness, and 43% for abdominal pain, with comparable results to those using metronidazole, however ciprofloxacin was slightly better tolerated^54^. This raises a concern regarding the Azole group, which is associated with side effects such as nausea, vomiting, a metallic taste, and peripheral neuropathy. Therefore, their use should be approached with caution.

In turn, a publication assessed the efficacy of norfloxacin and amoxicillin-clavulanic acid in patients with SIBO and chronic diarrhea. Norfloxacin reduced stool frequency by 45% and amoxicillin-clavulanic acid by 29%, both significantly better than placebo (*P*<0.01). The effects lasted an average of 6 days for both drugs. Hydrogen breath test results showed a two-thirds reduction in breath hydrogen volumes, with complete normalization in 30% of norfloxacin-treated and 50% of amoxicillin-clavulanic acid-treated patients^55^.

### Rifaximin

A growing body of evidence supports rifaximin, a nonabsorbable antibiotic, as the first-line treatment for SIBO, with recommendations from leading organizations^1^ ^,^ ^4^ ^,^ ^37^. These guidelines favor rifaximin due to its proven efficacy, non-systemic nature, and favorable safety profile.

In a retrospective study involving 443 patients, 53 tested positive for SIBO using a glucose hydrogen breath test. Rifaximin was prescribed to 78.4% of these patients at a dose of 550 mg three times daily for 14 days. The overall response rate was 47.4% for patients with hydrogen-positive breath tests and 80% for those with both hydrogen and methane positivity. These results highlight rifaximin’s effectiveness, particularly in hydrogen-positive SIBO, providing substantial relief for patients with this condition^56^.

Additionally, the TARGET trials included 1260 patients and randomized rifaximin to placebo to treat global IBS symptoms, IBS-related bloating, abdominal pain, and stool consistency. Rifaximin was shown to significantly decrease bloating in the two studies combined 40.2% vs 30.3%, *P*<0.001. The incidence of adverse events was similar in both groups^57^. A previous meta-analysis with 21 observational studies involving 874 patients showed the overall SIBO eradication was 59% in patients treated with rifaximin^58^. Rifaximin decreases inflammation and restore intestinal permeability through modulating the gut microbiota and increasing beneficial bacterial strains of the genera Lactobacillus and Bifidobacterium. TABLE 6 summarizes the main antibiotics and dosages used in the treatment of SIBO.

**Statement 7**: we propose using antibiotics as the primary treatment for symptomatic SIBO patients to eliminate bacterial overgrowth and alleviate symptoms.

### Special considerations

In underserved areas, where access to diagnostic tests for SIBO may be limited, a therapeutic trial of antibiotics can be a viable approach for patients with high clinical suspicion. In these cases, empirical treatment is initiated based on symptoms, without a diagnostic confirmation. This strategy ensures that patients receive timely care despite the lack of testing facilities, but it is essential to closely monitor treatment response to avoid unnecessary or prolonged antibiotic use.

In situations where rifaximin is unavailable, systemic antibiotics are generally recommended as alternatives. Supported by a larger body of scientific publications, ciprofloxacin or metronidazole are the preferred choices of our panel. However, the selection of systemic antibiotics should be individualized, taking into account any contraindications and the local availability of medications.

### Recurrence of SIBO

Recurrence of SIBO is a common challenge following antibiotic treatment. Despite initial successful eradication, this condition often reappears due to various underlying factors, including impaired gut motility, anatomical abnormalities, or the use of PPIs. These predisposing conditions complicate long-term management, as antibiotics alone do not address the root causes of the condition. Understanding the risk of recurrence and the contributing factors is crucial for optimizing treatment and preventing relapse.

A study conducted on 80 patients highlighted the recurrence rates of SIBO after rifaximin treatment. Following successful decontamination with rifaximin (1,200 mg/day for 7 days), glucose breath test (GBT) positivity recurrence rates were observed at 12.5% after 3 months, 27.5% after 6 months, and 43.7% after 9 months. The recurrence was significantly associated with older age (OR 1.09), a history of appendectomy (OR 5.9), and chronic PPI use (OR 3.52). 

Additionally, patients who experienced recurrent GBT positivity reported a significant increase in gastrointestinal symptoms such as bloating, abdominal pain, flatulence, and diarrhea, further underscoring the complexity of SIBO management^59^. Therefore, personalized treatment, along with efforts to eliminate associated risk factors, is essential to mitigate the risks related to repeated antibiotic therapy.

When gastrointestinal symptoms reappear, indicating a potential recurrence of SIBO, it is important to reassess the condition with a BT. If the test is positive and symptoms persist, initiating another course of antibiotic treatment may be necessary. However, there is currently a lack of definitive guidelines on the most effective treatment for recurrent SIBO. More research is needed to explore whether cyclic use of nonabsorbable antibiotics could offer a viable solution for long-term prevention of recurrence and improved symptom management^59^.

While there are many recommendations focused on addressing predisposing factors, especially in recurrent cases, there remains a lack of robust studies exploring pharmacological strategies to prevent SIBO recurrence. In this context, a retrospective study of 64 SIBO-positive IBS patients found that the nightly use of a low dose of tegaserod or erythromycin was significantly more effective than no treatment in preventing the recurrence of SIBO symptoms^60^.

### Probiotics

Probiotics are believed to have beneficial effects on the gut microbiota. However, few clinical studies have examined this option in SIBO therapy and its role is controversial because these studies lack consistency not only in the formulations used but also in the duration of treatment, populations assessed, and methods of diagnosing SIBO^1^ ^,^ ^18^ ^,^ ^61^ ^,^ ^62^.

More recently, a meta-analysis has examined 18 studies and reported that probiotics were associated with significantly increased clearance of SIBO compared with nonprobiotic therapy (six studies; relative risk, 1.6; 95%CI, 1.2-2.2), but the studies were mostly small and of poor quality. However, the associated SIBO-causing conditions were mixed, and although there may have been some improvement in symptoms such as abdominal pain, stool frequency was not impacted by probiotic therapy^63^. In addition, probiotics were not found to be efficacious for the prevention of SIBO. On the other hand, a controlled study showed that probiotics may actually cause SIBO and D-lactic acidosis leading to gas and bloating, and that withdrawal of probiotics combined with a course of antibiotics led to resolution of symptoms^19^.

Another recent randomized clinical trial compared two types of treatments for H_2_-SIBO and CH_4_-SIBO: one based on antibiotics and a low FODMAP diet (control group), and another using the same protocol, but with the addition of herbal supplements, probiotics, prebiotics, and glutamine (intervention group). Although the results showed no significant differences in the normalization of exhaled gas curves between groups, the patients in the intervention group showed an improved response in gastrointestinal symptoms, especially in CH_4_-SIBO^64^. However, a major limitation of the study was that the effects of the intervention group were not individualized.

In an open pilot clinical trial involving 40 patients with SIBO and systemic sclerosis, participants were divided into three groups: metronidazole (M), *Saccharomycis boulardii* (SB), and M + SB, for 2 months. Hydrogen was measured in parts per million using a hydrogen breath test to evaluate SIBO. After 2 months, SIBO was eradicated in 55% of the M + SB group, 33% of the SB group, and 25% of the M group. The SB and M + SB groups showed reductions in diarrhea, abdominal pain, and gas/bloating/flatulence, while the M group remained unchanged. Reductions in expired hydrogen at 45 to 60 minutes were as follows: M + SB 48% and 44%, M 18% and 20%, and SB 53% and 60% at the first and second months, respectively (*P*< 0.01). Adverse effects included epigastric burning and constipation in the M (53%) and M + SB (36%) groups, and flatulence/diarrhea in the SB group (22%). The study concluded that SB was effective as an adjuvant therapy in the treatment of SIBO^65^.

Finally, a systematic review analyzing five studies involving 266 patients with SIBO identified a significant variation in the use of probiotics as part of the therapy. Each study utilized different strains of probiotics. Two studies specifically examined the use of probiotics in combination with antibiotics, which limited the ability to draw definitive conclusions^66^.

In conclusion, studies used different methodologies in both breath testing and measurement of clinical symptoms, making it difficult to draw conclusions on SIBO eradication and symptom improvement across studies. 

**Statement 8**: the role of probiotics in SIBO treatment remains controversial, with limited evidence to support their routine use. Further research is needed to clarify their efficacy and potential impact on SIBO outcomes.

### Dietary changes

Diet is a modifiable factor that plays a crucial role in shaping the composition, diversity and stability of the gut microbiota^67^. A diet rich in fiber and plant-based foods, supplemented with prebiotics, and low in choline and fat is generally acknowledged to promote a healthy microbiota^67^. Conversely, a regimen deficient in fiber, rich in fermentable oligosaccharides, disaccharides, monosaccharides, and polyols (FODMAPs), and characterized by a high intake of omega-6 fatty acids, typical of a Western dietary pattern, is unfavorable and may predispose one to dysbiotic conditions^68^. In addition, it has been observed that patients with the diarrheal form of SIBO have a different gut microbiota composition compared to those with the constipated form^1^. On the other hand, the inability to recuperate permanently patients after any treatment for SIBO is particularly problematic. The number of patients with SIBO and its relapses is constantly increasing. Recurrent disease reduces the quality of life for patients^4^.

During the treatment, healthy nutrition should be a first-line priority. Nevertheless, such a diet is often insufficient to minimize gastrointestinal symptoms^69^. While a low-FODMAP diet is frequently used alongside therapeutic treatment and can help alleviate symptoms such as flatulence and bloating, it may also adversely affect microbiome diversity and, in this way, could be detrimental to long-term health^70^. For this reason, the restrictive phase of a low-FODMAP diet should not be followed for more than 6 weeks^69^. 

Vincenzi et al.^71^ evaluated 60 patients (35 female, 25 male, age range 20-65), divided into three groups: one treated with rifaximin plus a low-FODMAP diet, another with rifaximin plus a normal diet, and a third with placebo plus a low-FODMAP diet for 12 days. After the treatment period, both the rifaximin plus low-FODMAP diet group and the rifaximin plus normal diet group showed significant improvement in bloating and abdominal distension (*P*=0.000, and between 0.001 and 0.007 for other symptoms), while the third group showed a slight but not significant improvement. One-way ANOVA showed comparable symptom severity in the three groups pre-diet (*P*=0.215), but significant differences in symptoms after 12 days (*P*=0.000). The analysis revealed significant improvement in the first two groups compared to the third, and a trend toward improvement in the rifaximin plus normal diet group compared to the placebo plus low-FODMAP diet group. Similar results were observed for lactulose breath tests. There is no data on how a low-FODMAP diet, with or without rifaximin, affects bacterial overgrowth^69^.

Other elimination diets, such as gluten-free or lactose-free, are also used by patients with SIBO and may have a beneficial impact on gastrointestinal symptoms. However, similar to the low-FODMAP diet, they can have adverse effects on the gut microbiota. For this reason, elimination diets are never recommended without a valid indication^72^ ^,^ ^73^.

After eradication, eating habits could be a contributing factor to the development and relapse of SIBO, making adequate nutrition an important aspect of therapy. Products rich in polyphenols and soluble fiber, which act as prebiotics, have a beneficial effect on gut microbiota by selectively supporting bacterial growth^74^ ^,^ ^75^. However, there is currently insufficient data to definitively determine the optimal nutrition and supplementation strategies to prevent the recurrence and development of SIBO.

**Statement 9**: while the low FODMAP diet can help alleviate symptoms in the short term, long-term maintenance of such a restrictive diet is not recommended for SIBO patients.

### Intestinal methanogen overgrowth (IMO)

IMO is a newly proposed term to define the overproliferation of methanogens in both the small intestine and the colon^76^. First described in 1990, the methanogenic domain Archaea represents a phylogenetically distinct group of strictly anaerobic Euryarchaeota, whose energy metabolism is directed towards CH_4_ production from H_2_ and CO_2_ ^77^. Unlike bacteria, methanogens are less prevalent but play a complex role in human microbiome mutualism^78^. 

The Methanobacteriaceae family is comprised by three known phylotypes, such as Methanospaera stadmagnae, Methannobrevibacter oralis and Methanobrevibacter smithii, the last being the dominant one^76^. Over the past decade, methane has been significantly associated with intestinal motility disorders, functional constipation, and constipation-predominant IBS^79^ ^,^ ^80^. 

There is substantial evidence in the literature that elevated methane production augments ileal circular muscle activity, affects intestinal motor function, and slows small intestinal transit^81^ ^,^ ^82^. In addition, some authors have shown that methane not only significantly increased ileal contraction amplitudes in guinea pigs, but also were inhibited by atropine infusion, suggesting a dependent cholinergic pathway of the enteric nervous system^83^. Until now, the exact mechanism on how methane acts in the small intestine is not completely understood.

In clinical practice, the diagnosis is based on a positive breath test as a surrogate marker for IMO, as small bowel culture is an invasive method, requires sedation, is expensive and has limited availability. Recently, the North American Consensus defined a positive test as a rise over baseline in breath methane concentrations of equal or greater than 10 ppm^83^.

Antibiotic therapy should be introduced only after confirming IMO via a breath test and should not be empirically initiated to treat constipation. The first-line treatment for IMO is a combination of neomycin (500 mg twice daily) and rifaximin (550 mg three times daily) for 14 days. Other antibiotic regimens, such as rifaximin with metronidazole, amoxicillin-clavulanate, or ciprofloxacin and metronidazole, have also been studied in this population^1^. Low et al. retrospectively reviewed the antibiotics regimen in 74 patients diagnosed with IMO. Complete eradication of methane occurred in 87% of subjects treated with rifaximin and neomycin, compared with 33% of subjects in the neomycin group and 28% of subjects in the rifaximin group (*P*=0.001)^84^. 

## CONCLUSION

In this position paper, we have outlined the current understanding of SIBO with a specific focus on its diagnosis and treatment within the Brazilian healthcare context. SIBO remains a challenging condition to diagnose due to the overlap of symptoms with other gastrointestinal disorders, yet breath testing and jejunal aspirates offer viable diagnostic pathways. Rifaximin continues to be the first-line treatment for SIBO due to its non-systemic nature and efficacy. In cases where rifaximin is unavailable, systemic antibiotics such as ciprofloxacin and metronidazole serve as appropriate alternatives.

Addressing the challenges of diagnostic accessibility, particularly in underserved areas, remains critical. As we continue to explore new diagnostic tools, refine treatment options, and emphasize the importance of addressing risk factors for recurrence, ongoing research and standardized protocols will be essential in optimizing the management of SIBO in Brazil. Further studies are needed to expand our understanding of this condition, particularly within the unique context of the Brazilian population.

## FUTURE DIRECTIONS FOR SIBO MANAGEMENT IN BRAZIL

### Epidemiology and characteristics:

- Identify gaps in the understanding of SIBO within the Brazilian population.
- Conduct population-based studies to determine the prevalence and specific risk factors in Brazil.

### Improving test availability:

- Expand access to breath tests, which are currently limited, especially within the public healthcare system in Brazil, and emphasize the importance of incorporating methane breath tests into clinical practice to improve diagnostic accuracy.

### Treatment evaluation:

- Conduct comparative studies of antimicrobial treatments to assess efficacy and safety within the Brazilian context.
- Investigate non-pharmacological approaches, such as probiotics and dietary interventions, to personalize and optimize treatment.

### Enhancing care in the Brazilian healthcare system

- Promote continuous education and training for physicians and healthcare professionals on the management of SIBO, particularly in underserved regions with a shortage of specialists.
- Facilitate the inclusion of treatments and diagnostic tests within the Brazilian Public Health System (SUS), aiming for equitable access to care.
